# Structure-Activity Determinants in Antifungal Plant Defensins MsDef1 and MtDef4 with Different Modes of Action against *Fusarium graminearum*

**DOI:** 10.1371/journal.pone.0018550

**Published:** 2011-04-13

**Authors:** Uma Shankar Sagaram, Raghoottama Pandurangi, Jagdeep Kaur, Thomas J. Smith, Dilip M. Shah

**Affiliations:** Donald Danforth Plant Science Center, Saint Louis, Missouri, United States of America; University of Missouri-Kansas City, United States of America

## Abstract

Plant defensins are small cysteine-rich antimicrobial proteins. Their three-dimensional structures are similar in that they consist of an α-helix and three anti-parallel β-strands stabilized by four disulfide bonds. Plant defensins MsDef1 and MtDef4 are potent inhibitors of the growth of several filamentous fungi including *Fusarium graminearum*. However, they differ markedly in their antifungal properties as well as modes of antifungal action. MsDef1 induces prolific hyperbranching of fungal hyphae, whereas MtDef4 does not. Both defensins contain a highly conserved γ-core motif (GXCX_3–9_C), a hallmark signature present in the disulfide-stabilized antimicrobial peptides, composed of β2 and β3 strands and the interposed loop. The γ-core motifs of these two defensins differ significantly in their primary amino acid sequences and in their net charge. In this study, we have found that the major determinants of the antifungal activity and morphogenicity of these defensins reside in their γ-core motifs. The MsDef1-γ4 variant in which the γ-core motif of MsDef1 was replaced by that of MtDef4 was almost as potent as MtDef4 and also failed to induce hyperbranching of fungal hyphae. Importantly, the γ-core motif of MtDef4 alone was capable of inhibiting fungal growth, but that of MsDef1 was not. The analysis of synthetic γ-core variants of MtDef4 indicated that the cationic and hydrophobic amino acids were important for antifungal activity. Both MsDef1 and MtDef4 induced plasma membrane permeabilization; however, kinetic studies revealed that MtDef4 was more efficient in permeabilizing fungal plasma membrane than MsDef1. Furthermore, the *in vitro* antifungal activity of MsDef1, MsDef1-γ4, MtDef4 and peptides derived from the γ-core motif of each defensin was not solely dependent on their ability to permeabilize the fungal plasma membrane. The data reported here indicate that the γ-core motif defines the unique antifungal properties of each defensin and may facilitate *de novo* design of more potent antifungal peptides.

## Introduction

Plants are sessile organisms that are continually challenged by microbial pathogens during their life cycle. To ward off pathogen attack, plants produce a number of cationic antimicrobial peptides. These include defensins that are one of the largest families of antimicrobial peptides found in plants. These basic, cysteine-rich, proteins are 45 to 54 amino acids in length and share significant structural homology with defensins from insects, mollusks and mammals [1]. All plant defensins contain an invariant tetradisulfide array and share a common cysteine-stablized α/β structure (CSα/β) composed of three antiparallel β-strands and one α-helix. Despite their structural similarity, the amino acid sequences of plant defensins are highly diverse [2], [3]. This variation in primary sequences may account for different functions attributed to plant defensins including antibacterial activity, zinc tolerance, proteinase and α-amylase inhibitory activity, ion channel blocking activity [4] as well as pollen tube growth arrest, burst and sperm discharge [5].

A large number of cationic plant defensins exhibit inhibitory activity against filamentous fungi *in vitro* and in transgenic plants [2], [3], [4], [6], [7], [8]. Because of their potent *in vitro* antifungal activity, plant defensins have the potential to be used as antifungal agents in transgenic crops. A growing body of evidence suggests that plant defensins with highly diverse primary structures inhibit the growth of target fungi via different modes of action [9], [10], [11]. For example, RsAFP2 from *Raphanus sativus* and DmAMP1 from *Dahlia merckii* bind to distinct sphingolipids in membranes of fungi and this interaction with sphingolipids is required for their antifungal activity [12], [13], [14]. Other plant defensins like MsDef1 and ZmES4 likely act on ion channels [5], [15].

MsDef1, a 45-amino acid protein from the seed of *Medicago sativa*, inhibits the growth of a filamentous fungus, *Fusarium graminearum*, at micromolar concentrations. MtDef4 is a 47-amino acid protein that is expressed constitutively and in response to biotic and abiotic stress in many tissues of a model legume, *M. truncatula*. Based on their effects on the morphology of fungal hyphae, antifungal plant defensins are divided into two different subgroups, referred to as morphogenic and nonmorphogenic. Morphogenic defensins inhibit hyphal growth with a concomitant increase in hyphal branching, whereas nonmorphogenic defensins inhibit hyphal growth without causing marked morphological alterations [16], [17]. MsDef1 is a morphogenic defensin that induces extensive hyperbranching of fungal hyphae, whereas MtDef4 is a nonmorphogenic defensin that does not induce hyperbranching [18]. MtDef4 is more potent against *F. graminearum* than MsDef1 [18]. Two lines of evidence indicate that MsDef1 and MtDef4 have different modes of antifungal action. First, insertional mutants of *F. graminearum* that were isolated as being hypersensitive to MsDef1 exhibit no change in their sensitivity to MtDef4 [18]. The analysis of these mutants revealed two mitogen-activated protein kinase signaling cascades that were required for the protection of the fungus from the toxic effects of MsDef1. Second, a mutant depleted in the plasma membrane sphingolipid glucosylceramide, designated ΔFg*gcs1*, was found to be highly resistant to MsDef1, but retained the wild-type sensitivity to MtDef4 [19].

Little is known about the structural determinants of the *in vitro* antifungal activity of MsDef1 and MtDef4. Since all plant defensins whose 3-D structures have been determined have a similar backbone, any differences in their antifungal activities and specificities are likely to arise primarily from differences in the amino acid composition and charge distribution of solvent-exposed loops. The calculated net positive charge of +6 for MtDef4 is significantly higher than the calculated net positive charge of +3 for MsDef1. Also, the predicted solvent exposed γ-core (see results) of MtDef4 has significantly higher positive charge than that of MsDef1. We previously reported that the carboxy-terminal amino acid sequence (AA_31_ to AA_45_) was a major determinant of the *in vitro* antifungal activity of MsDef1 and that R38Q mutation significantly reduced its antifungal activity [15]. This sequence spans the β2 and β3 strands and the interposed loop on the homology-based 3-D structure of MsDef1 [20], previously referred to as AlfAFP [21]. It also contains the γ-core motif GXC(X_3–9_)C conserved among disulfide-containing antimicrobial peptides. This motif is characterized by the presence of two antiparallel β strands with an interposed loop that has a net cationic charge and participates in one to four disulfide bonds [22]. The γ-core motif is conserved in all antifungal plant defensins including MsDef1 and MtDef4.

In this study, we have identified and functionally characterized the contributions of the γ-core motifs to the antifungal activity of MsDef1 and MtDef4. We show that the MsDef1-γ4 variant in which γ-core motif of MsDef1 was replaced with that of MtDef4 behaved like a nonmorphogenic defensin with antifungal activity similar to that of MtDef4. The chemically synthesized peptides that contained the γ-core motif plus the carboxy-terminal 6 amino acids of each defensin also exhibited antifungal activity that was less potent than that of a full length defensin. Importantly, the γ-core motif of MtDef4 alone was sufficient for antifungal activity, whereas that of MsDef1 was not. We further show that the positively charged amino acids and hydrophobic side chains present in the γ-core loop are important for the antifungal activity of MtDef4. Furthermore, MsDef1, MsDef1-γ4 and MtDef4 markedly differed in their ability to permeabilize fungal plasma membrane, but membrane permeabilization was not the sole determinant of antifungal activity.

## Results

### MsDef1 and MtDef4 contain highly conserved γ-core motifs

**Figure 1:**
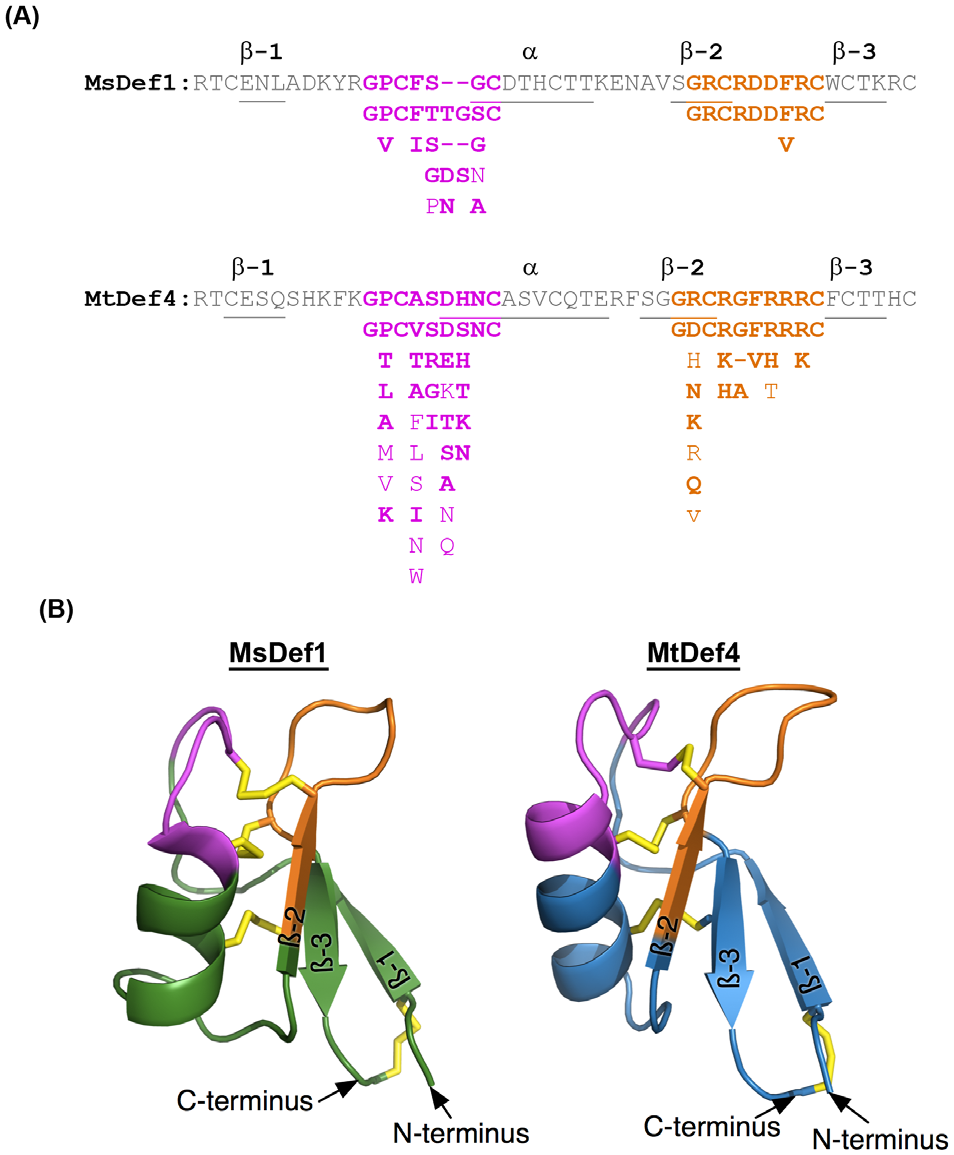
Highly conserved structural motifs and homology-based three-dimensional structures of plant defensins MsDef1 and MtDef4. (A) Amino acid sequences of MsDef1 and MtDef4 showing highly conserved α-core and γ-core motifs. The consensus α-core (pink color) and γ-core (orange color) sequences were created using 16 homologs of MsDef1 and 28 homologs of MtDef4. For both sequences, conserved amino acids of the core regions are listed underneath the respective motifs with highly conserved amino acids on the top followed by less conserved ones. α-helix and β-strands are underlined. (B) Homology-based models of MsDef1 and MtDef4. The α-core motif is indicated in pink color; the γ-core motif is indicated in orange color and the four disulfide bridges are indicated in yellow color. Models were developed using the I-TASSER website for protein structure and function predictions as described in Materials and Methods.

Previous structure-function analysis of disulfide-containing antimicrobial peptides from phylogenetically diverse organisms identified a conserved three-dimensional γ-core motif with a consensus sequence GXC(X_3–9_)C [22]. Analysis of the MsDef1 and MtDef4 primary sequences revealed the presence of a γ-core motif encompassing β2–β3 loops of their homology predicted 3-D structures (Figure 1). Several plant defensin sequences deposited in the PhytAMP database (http://phytamp.pfba-lab-tun.org/main.php) also contain the consensus γ-core motif. A search of the NCBI database using Blast P [23] resulted in the identification of 16 legume-specific plant defensin sequences sharing high sequence homology with MsDef1 (Figure S1A). In contrast, defensin sequences sharing high sequence homology with MtDef4 are widespread throughout the plant kingdom (Figure S1B). CLUSTAL W alignment [24] of 18 amino acid sequences of the homologs of MsDef1 identified a consensus γ-core motif with the sequence ‘GXCRDD(F/V)RC’ (Figure 1A), whereas similar analysis of 28 MtDef4 homologs identified a consensus γ-core motif with the sequence ‘GXC(R/H/K)(G/A/_)(F/V)(R/H/T)R(R/K)C’ (Figure 1A). The γ-core motifs of MsDef1 and MtDef4 are GRCRDDFRC and GRCRGFRRRC, respectively. Based on their predicted structures (Figure 1B), both MsDef1 and MtDef4 γ-core motifs connect the two anti-parallel β2 and β3 strands and contain an interposed loop. Each γ-core motif has a net positive charge and a hydrophobic amino acid. The analysis of the amino acid sequences of MsDef1 and MtDef4 homologs also revealed the presence of another motif containing the GXC sequence. We have designated this motif as α-core motif with a consensus sequence GXC(X_3–5_)C. The α-core motifs of MsDef1 and MtDef4 are GPCFSGC and GPCASDHNC, respectively (Figure 1A). This motif resides in the β1 strand-α-helix loop and contains part of the α-helix of each defensin. This motif however lacks the hairpin structure of the γ-core motif.

### The γ-core substitution variant of MsDef1 containing the γ-core motif of MtDef4 has significantly enhanced antifungal activity and becomes a nonmorphogenic defensin

**Figure 2:**
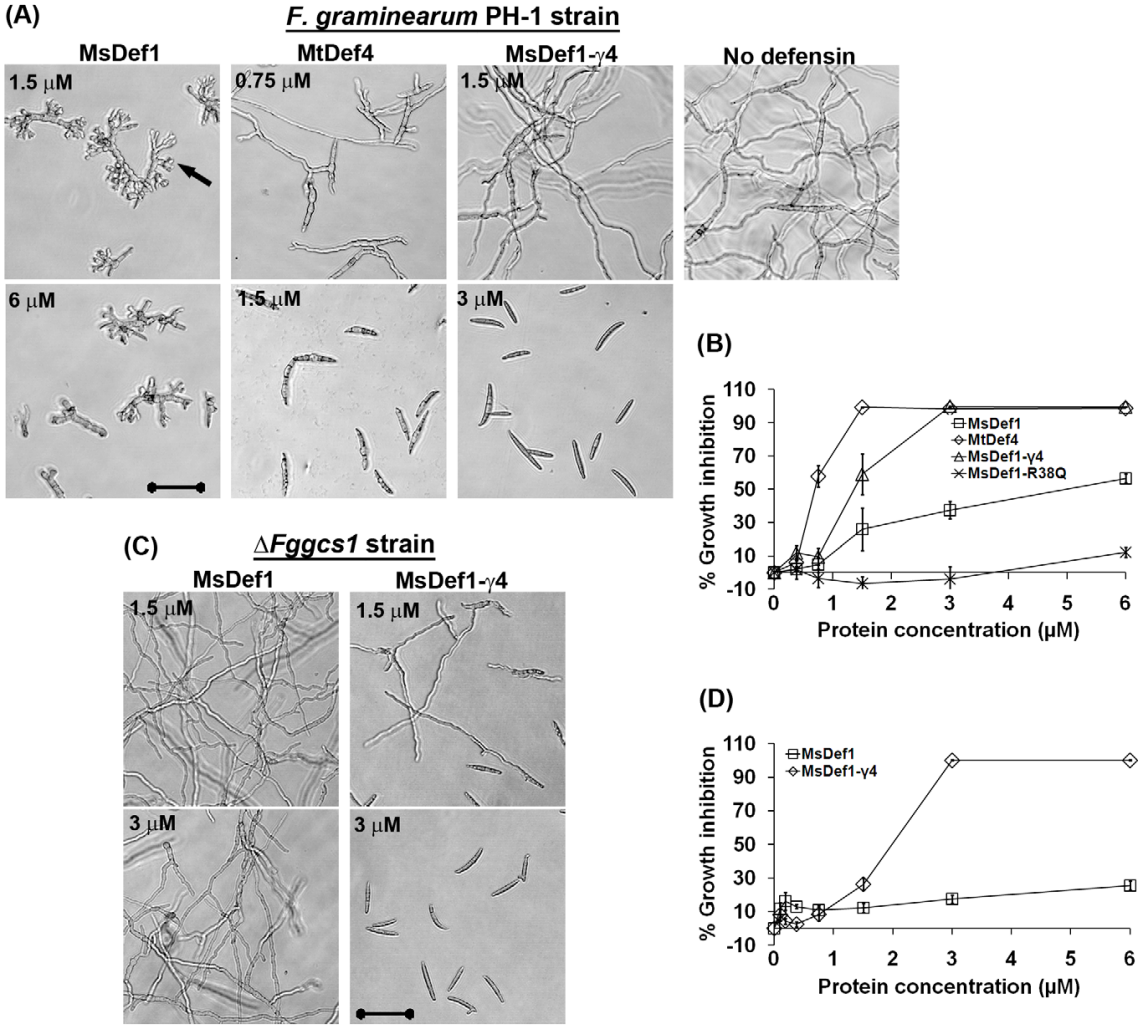
MsDef1 γ-core substitution variant, MsDef1-γ4, exhibits antifungal activity similar to that of MtDef4 against the wild-type *F. graminearum* PH-1 and the MsDef1-resistant mutant *ΔFggcs1* lacking glucosylceramide. (A) Images showing the inhibition of conidial germination and hyphal growth at different concentrations of MsDef1, MtDef4 and the variant MsDef1-γ4. Images were taken after 16 hours of incubation of PH-1 conidia with defensins. Bar = 50 µm. Hyperbranching of hyphae in the presence of MsDef1 is indicated with black arrow. (B) Quantitative assessment of the antifungal activity of MsDef1, MtDef4 and the variant MsDef1-γ4 against PH-1 strain. (C) Images showing the inhibition of conidial germination and hyphal growth of MsDef1-resistant mutant *ΔFggcs1* lacking glucosylceramaide at different concentrations of MsDef1 and the variant MsDef1-γ4. Images were taken after 16 hours of incubation of *ΔFggcs1* conidia with defensins. Bar = 50 µm. (D) Quantitative measurement of the antifungal activity of MsDef1 and its γ-core substitution variant MsDef1-γ4 against *ΔFggcs1* strain. Values are means of three replications. Error bars indicate standard deviations.

Defensins containing the γ-core motif identical to that of MtDef4 are widely distributed in the plant kingdom, whereas those containing the γ-core motif identical to that of MsDef1 are only found in legumes. We therefore replaced the γ-core motif of MsDef1 with that of MtDef4 to determine what effect this replacement would have on MsDef1's ability to inhibit the growth of *F. graminearum* and to induce hyperbranching of hyphae in this fungus. A chimera of MsDef1 containing the γ-core motif of MtDef4 was created by replacing the RDDFR sequence with RGFRRR and designated MsDef1-γ4. The MsDef1-γ4 variant has significantly higher net positive charge (+7) when compared to MsDef1 that has a net charge of +3 (Table 1). Mass spectrometric analysis demonstrated that MsDef1-γ4 had expected mass and formed four disulfide bonds (data not shown). The antifungal activity of this variant was compared to that of MsDef1 and MtDef4 microscopically as well as spectrophotometrically. Microscopic observations after overnight incubation of fungal macroconidia (conidia) indicated that MsDef1-γ4 exhibited antifungal activity at concentrations as low as 0.375 µM. At 1.5 µM, it inhibited 59±12% of fungal growth, which is twice as effective as MsDef1 (26±12%) (Figures 2A and B). MtDef4 inhibited conidial germination completely (100% growth inhibition) at this concentration (Figure 2A). At 3 µM, MsDef1-γ4 completely inhibited the germination of conidia. In contrast, conidia were able to germinate and grow even in the presence of 6 µM MsDef1. MsDef1 caused only 38±5% and 57±3% growth inhibition at 3 µM and 6 µM, respectively (Figure 2B). Thus, the MsDef1-γ4 variant exhibited significantly higher antifungal activity than MsDef1 and was almost as potent as MtDef4. As reported previously [15], the MsDef1-R38Q variant showed no antifungal activity in this experiment and may as such act as a negative control (Figure 2B). Significantly, hyperbranching of fungal hyphae, which is a typical morphological response of *F. graminearum* to MsDef1, was not observed in the presence of MsDef1-γ4 variant (Figure 2A).

**Table 1:** List of amino acid sequences, net charge and growth inhibitory concentrations against *F. graminearum* PH-1.

Name		@Type	#Charge	$IC_50_	&IC_100_
**A. Full length proteins with mixed αβ conformation**					
MsDef1	RT**C**ENLADKYRGP**C**FSG**C**DTH**C**TTKENAVSGR**C**RDDFR**C**W**C**TKR**C**	Mixed αβ	+3	2–4	>6
MtDef4	RT**C**ESQSHKFKGP**C**ASDHN**C**ASV**C**QTERFSGGR**C**RGFRRR**C**F**C**TTH**C**	Mixed αβ	+6	0.75–1	1.5–2.5
MsDef1-γ4*	RT**C**ENLADKYRGP**C**FSG**C**DTH**C**TTKENAVSGR**C***RGFRRR***C**W**C**TKR**C**	Mixed αβ	+7	1.3–1.5	3–4
MsDef1-R38Q*	RT**C**ENLADKYRGP**C**FSG**C**DTH**C**TTKENAVSGR**C**RDDF*Q***C**W**C**TKR**C**	Mixed αβ	+2	>6	>24
**B. Peptides corresponding to original sequences from MsDef1 or MtDef4**					
GMA1-C	GR**C**RDDFR**C**W**C**TKR**C**	Unknown	+3	14	24
GMA4-C	GR**C**RGFRRR**C**F**C**TTH**C**	Unknown	+5	3	6
GMA1	GR**C**RDDFR**C**	Unknown	+1	>192	>192
GMA4	GR**C**RGFRRR**C**	Unknown	+5	3	6
GMA1-L	RDDFR	Unknown	0	>96	>96
GMA4-L	RGFRRR	Unknown	+4	4	12
ALP1	GP**C**FSG**C**	Unknown	0	>48	>48
ALP4	GP**C**ASDHN**C**	Unknown	−1	>48	>48
**C. Variant peptides of GMA4-L**					
GMA4-L1	RGARRR	Unknown	+4	>96	>96
GMA4-L2	RGFARR	Unknown	+3	>96	>96

@ Type = Protein/peptide conformation; Mixed αβ means, structure includes both α-helix and β-strand conformation. Unknown = peptide structure not determined.

# Net charge of the peptide was determined using Biochemistry Online- http://vitalonic.narod.ru/biochem/index_en.html.

$ Amount of protein (µM) required for inhibiting 50% growth of *F. graminearum*.

& Amount of protein (µM) required to inhibit *F. graminearum* conidial germination completely.

*Replaced amino acid(s) are italicized.

Cysteines are indicated in bold font.

We have previously characterized a mutant strain of *F. graminearum*, *Δ*Fg*gcs1*, lacking sphingolipid glucosylceramide (GlcCer) in the plasma membrane and demonstrated that it is resistant to MsDef1, but not to MtDef4 [19]. Interestingly, the *Δ*Fg*gcs1* strain was highly sensitive to MsDef1-γ4 at concentrations that were insufficient for MsDef1 to be effective (Figure 2C). At 1.5 µM, the MsDef1-γ4 variant, but not MsDef1, inhibited the growth of the *Δ*Fg*gcs1* strain. MsDef1-γ4 variant completely inhibited conidial germination of the *Δ*Fg*gcs1* strain at 3 µM, whereas MsDef1 caused only 16±2% inhibition at 3 µM and 23±2% inhibition at 6 µM (Figure 2D).

Together, these results suggest the γ-core motif of MsDef1 contains the major determinants of its morphogenicity and likely interacts with GlcCer as part of MsDef1's antifungal action.

### γ-core motif is a vital structural component of MsDef1 that affects hyphal tip growth

**Figure 3:**
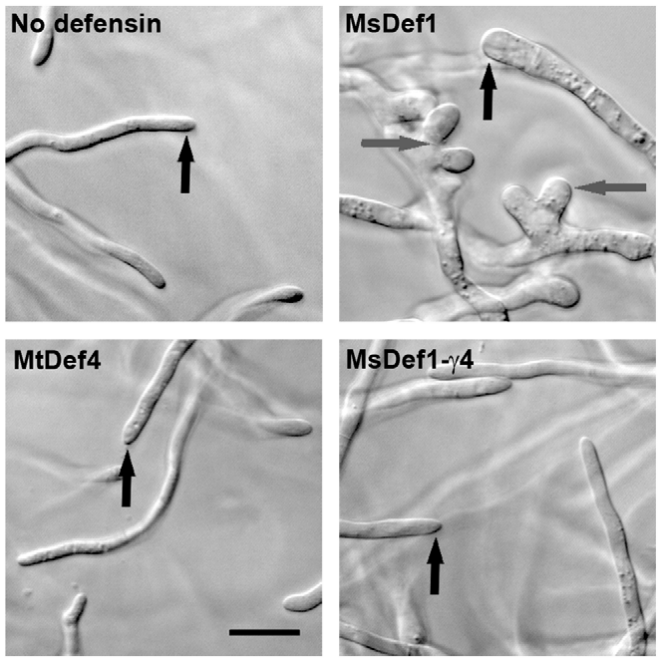
MsDef1 induces significant changes in the growing hyphal tip morphology of *F. graminearum*, but MtDef4 and MsDef1-γ4 do not. Images showing the hyphal tips of untreated fungal hyphae and those of fungal hyphae treated with IC_100_ concentrations of MsDef1 (12 µM), MtDef4 (1.5 µM) and the variant MsDef1-γ4 (3 µM). Hyphal tips of fungal hyphae treated with MtDef4 and MsDef1-γ4 appear similar to those of untreated fungal hyphae, whereas hyphal tips of fungal hyphae treated with MsDef1 appear swollen. Black arrows point to the hyphal tips. The division of hyphal tips in the presence of only MsDef1 is indicated with gray arrows. Bar = 20 µm. All images were taken between 7–8 hours after incubation of PH-1 germlings with defensins.

Since the hyperbranching of fungal hyphae was not observed in *F. graminearum* treated with the MsDef1-γ4 variant, it was speculated that the γ-core motif of MsDef1 affected hyphal tip growth. To test this hypothesis, fungal hyphae were treated with complete inhibitory concentrations (Table 1) of MsDef1 or MtDef4 or MsDef1-γ4 and their effects on hyphal morphology were monitored and compared with the morphology of untreated hyphae. At 4 hours, the hyphae treated with MsDef1 appeared swollen with conspicuous bulges at the tips. In contrast, the hyphae treated with MtDef4 and MsDef1-γ4 appeared similar to untreated control hyphae. After 7–8 hours of treatment, the hyphae treated with MsDef1 remained swollen and the tips started to branch dichotomously (Figure 3). More than 90% of the tips treated with MsDef1 had apparent swelling morphology or were in the process of division, where as no difference was noticed in morphology of hyphae treated with either MtDef4 or the MsDef1-γ4 variant or the untreated control hyphae (Figure 3). Based on these observations, we speculate that the γ-core region of MsDef1 directly or indirectly interacts with the molecular determinants of hyphal tip growth thus resulting in hyperbranching of fungal hyphae. The substitution of this motif with that of MtDef4 in the MsDef1-γ4 variant likely prevents the proposed interaction resulting in the lack of hyperbranching effect.

### MsDef1, MsDef1-γ4 variant and MtDef4 permeabilize the plasma membrane of *F. graminearum*, but with different intensities

**Figure 4:**
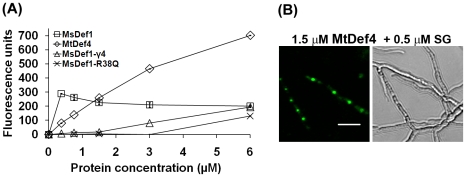
MsDef1, MtDef4 and the MsDef1 variants, MsDef1-γ4 and MsDef1-R38Q, permeabilize the plasma membrane of *F. graminearum*, but with different intensities. (A) Quantitative measurement of Sytox Green (SG) uptake by *F. graminearum* hyphae treated with MsDef1, MsDef1-γ4, MtDef4, and MsDef1-R38Q (488 excitation; 540 emission (530 cut-off)) for 5 hours, (B) Fluorescence image of SG binding to the nuclei of *F. graminearum* following treatment with MtDef4. Bar = 20 µm.

One characteristic feature of many cationic antimicrobial peptides is their ability to permeabilize the plasma membrane of their target organisms. Plant defensins have been previously shown to permeabilize fungal plasma membrane [25], [26]. Because of their different modes of antifungal action and morphological effects on *F. graminearum*, MsDef1, MsDef1-γ4 and MtDef4 were examined for their ability to permeabilize the fungal plasma membrane using the fluorometric SYTOX Green (SG) (Molecular Probes Inc., OR), a dye which is only taken up by cells with compromised plasma membrane. Fluorescence of this dye increases >500-fold upon binding to nucleic acids thus allowing quantitative analysis and fluorescence microscopy [27]. Permeabilization was measured by fluorescence microscopy and spectrophotometry using SG uptake assay. SG uptake was monitored at regular intervals (15 minutes, 30 minutes, 1 hour, 2 hours, 3.5 hours, 5 hours and 6 hours). The SG uptake increased during exposure to defensins and started to plateau at 5 hours. Therefore, the fluorescence at 5 hours represents the maximum effect of the defensins and is correlated with the concentration of defensins in Figure 4. SG uptake steadily increased with an increase in MtDef4 concentration, whereas MsDef1 induced a rapid uptake of SG at low concentrations (<1 µM) that became plateau at 1 to 6 µM. At a concentration of 6 µM, MtDef4 treated hyphae emitted a maximum fluorescence of 702±10 units at 5 hours (Figure 4A), whereas MsDef1 treated hyphae had a maximum fluorescence of 304±5 units at 2 hours.

The defensin-induced uptake of SG was also examined using fluorescence microscopy. SG uptake by *F. graminearum* hyphae started within 5 minutes of the addition of MsDef1 and MtDef4. SG entered and bound to the nuclei of hyphae treated with both MsDef1 (not shown) and MtDef4 (Figure 4B) within 2 hours. No significant differences were observed in the SG uptake in hyphae treated with MsDef1 at concentrations of 0.375, 0.75, 1.5, 3.0 and 6.0 µM. In contrast, MtDef4-induced SG uptake increased in a concentration dependant manner. At higher concentrations, a large number of MtDef4-treated hyphae were affected as compared to the MsDef1-treated hyphae (Figure S2). With both MsDef1 and MtDef4, heavily affected hyphae appeared much darker and granular and the SG appeared disseminated inside the hyphae (data not shown). However, it is also important to note that not all hyphae treated with either MsDef1 or MtDef4 were uniformly affected. These studies suggest that MtDef4 induces plasma membrane damage to a much greater extent than MsDef1. This is also consistent with the more potent *in vitro* antifungal activity of MtDef4 than that of MsDef1.

To better understand the relationship between plasma membrane permeabilization and antifungal activity of MsDef1, SG uptake by hyphae treated with two variants of MsDef1 were examined; MsDef1-R38Q variant with significantly reduced antifungal activity [15] and MsDef1-γ4 substitution variant with antifungal activity nearly as potent as that of MtDef4. As expected, MsDef1-R38Q induced little or no uptake of SG by fungal hyphae (Figure 4A). Surprisingly, MsDef1-γ4 variant also induced little (maximum 82±2 units) uptake of SG even at a concentration of 3 µM, a concentration that is sufficient for 100% growth inhibition (Figure 4A).

Taken together, our data indicated that MsDef1, MsDef1-γ4 and MtDef4 affected the permeability of fungal plasma membrane, but their intensities of permeabilization were significantly different. Importantly, the degree of permeabilization did not correlate well with the antifungal potency.

### Synthetic peptides containing the γ-core motifs plus carboxy-terminal 6 amino acids of MsDef1 and MtDef4 inhibit the growth of *F. graminearum*

**Figure 5:**
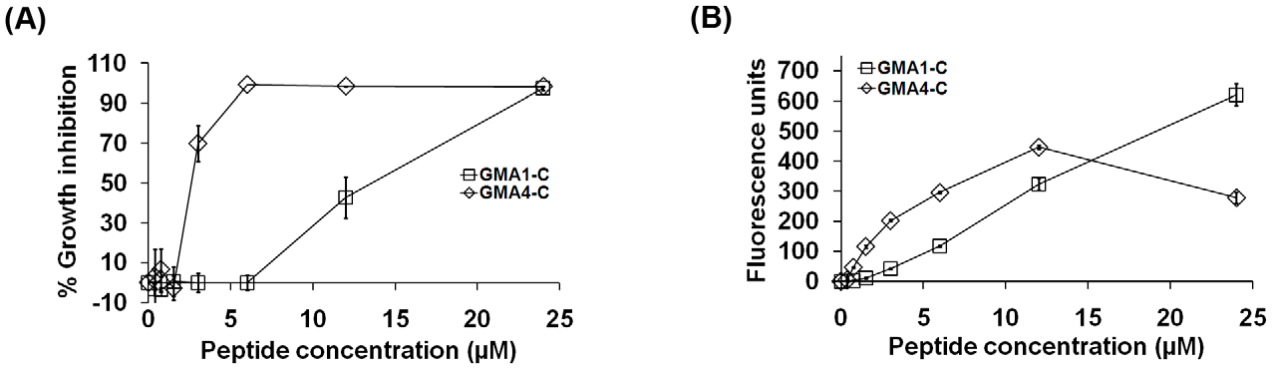
GMA1-C and GMA4-C peptides containing the γ-core sequences plus the carboxy-terminal 6 amino acids of MsDef1 and MtDef4 exhibit antifungal activity and induce plasma membrane permeabilization in *F. graminearum*. (A) Quantitative measurement of fungal growth inhibition caused by treatment with GMA1-C and GMA4-C. Values are means of at least three replications. Error bars indicate standard deviations, (B) Quantitative measurement of SG uptake by *F. graminearum* PH-1 hyphae treated with GMA1-C or GMA4-C.

In order to determine the contribution of the γ-core motif to the antifungal activity of MsDef1 and MtDef4, two chemically synthesized peptides, GMA1-C and GMA4-C, containing the γ-core motif sequence and six carboxy-terminal amino acids of MsDef1 and MtDef4, respectively (Table 1) were tested for antifungal activity. GMA1-C and GMA4-C were 15 and 16 amino acids in length, respectively. Each peptide contained four cysteine residues with no S-S bonds as determined by mass spectrometry (data not shown). At 16 hours after incubation of conidia with peptides, inhibition of fungal growth was clearly evident at 6 µM of GMA1-C and 1.5 µM of GMA4-C (Figure S3). However, these concentrations did not have any effect on fungal growth after 36 hours. Quantitation of fungal growth spectrophotometrically indicated that the inhibition of fungal growth was 43±10% at 12 µM of GMA1-C peptide, whereas the inhibition in the presence of 3 µM GMA4-C peptide was 70±9% (Figure 5A). Both GMA1-C and GMA4-C peptides completely inhibited the germination of conidia at 24 and 6 µM, respectively (Figure S3). It is important to note, however, that the GMA1-C peptide did not cause hyperbranching of fungal hyphae, a hallmark feature of MsDef1, indicating that the carboxy-terminal 15 amino acid sequence containing the γ-core motif alone is not the sole determinant of the morphogenicity of MsDef1 [18].

GMA1-C and GMA4-C peptides also induced the uptake of SG, indicating that they permeabilized the fungal plasma membrane (Figure 5B). At 12 µM, GMA4-C was more effective in inducing SG uptake than GMA1-C. However, at 24 µM, GMA1-C-induced SG uptake was twice that induced by GMA4-C. This again demonstrates that there is not a direct relationship between plasma membrane disruption and antifungal activity.

### MtDef4 γ-core motif alone exhibits antifungal activity, but MsDef1 γ-core motif does not

**Figure 6:**
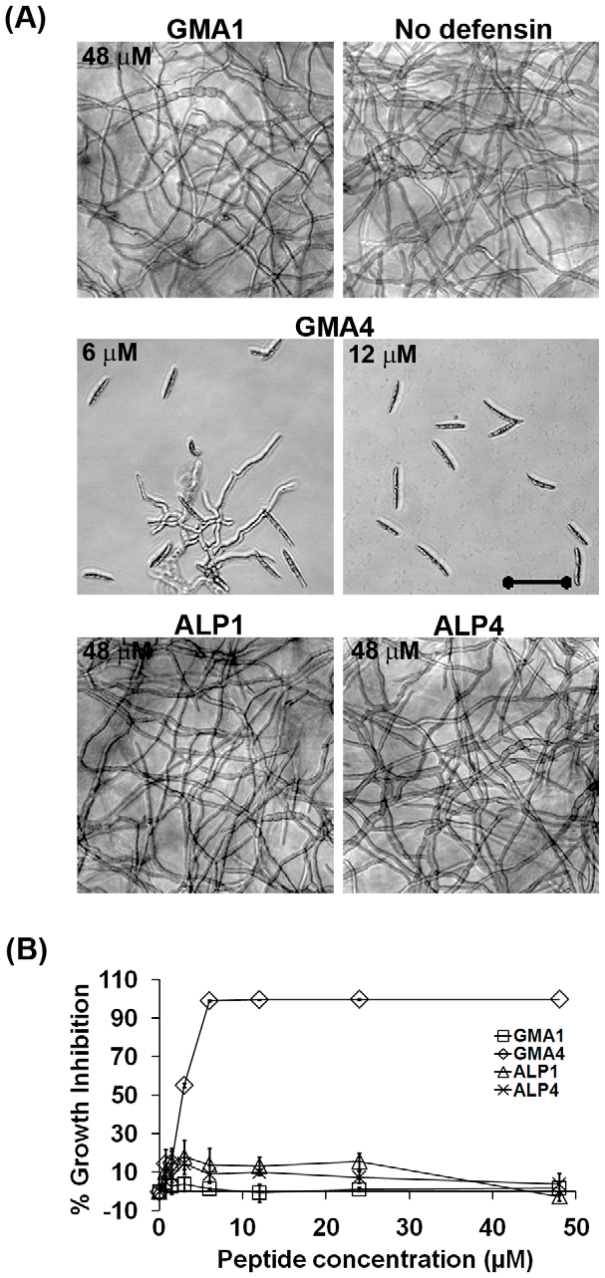
GMA4 peptide containing only the γ-core sequence of MtDef4 exhibits antifungal activity, but the GMA1 peptide containing only the γ-core sequence of MsDef1 and the α-core peptides, ALP1 & ALP4, of both defensins do not. (A) GMA4 shows potent antifungal activity at concentration as low as 6 µM, while GMA1 as well as ALP1 and ALP4 show no antifungal activity even at 48 µM. (B) Quantitative measurement of fungal growth inhibition caused by treatment with GMA1, GMA4, ALP1 and ALP4. Values are means of at least three replications. Error bars indicate standard deviations.

Two peptides, GMA1 (GRCRDDFRC) and GMA4 (GRCRGFRRRC) (Table 1), representing the only γ-core motif of each defensin were examined for antifungal activity. As shown in Figure 6A and Figure 6B, GMA4 exhibited antifungal activity at 6 µM and completely inhibited conidial germination at 12 µM. In contrast, GMA1 even at a concentration of 96 µM failed to show any antifungal activity. From these results, it is apparent that, while MtDef4 γ-core motif alone is sufficient for antifungal activity, the MsDef1 γ-core is not.

As previously described, MsDef1 and MtDef4 contain the α-core motif with a consensus sequence of GXC(X_3–5_)C. The α-core motif of each defensin was chemically synthesized and tested for antifungal activity. Both ALP1 (GPCFSGC) and ALP4 (GPCASDHNC) peptides were totally inactive at all concentrations tested (Figures 6A and 6B) indicating that the α-core motifs of MsDef1 and MtDef4 alone do not exhibit antifungal activity.

### RGFRRR hexapeptide is a major contributor to the antifungal activity of the γ-core motif of MtDef4

**Figure 7:**
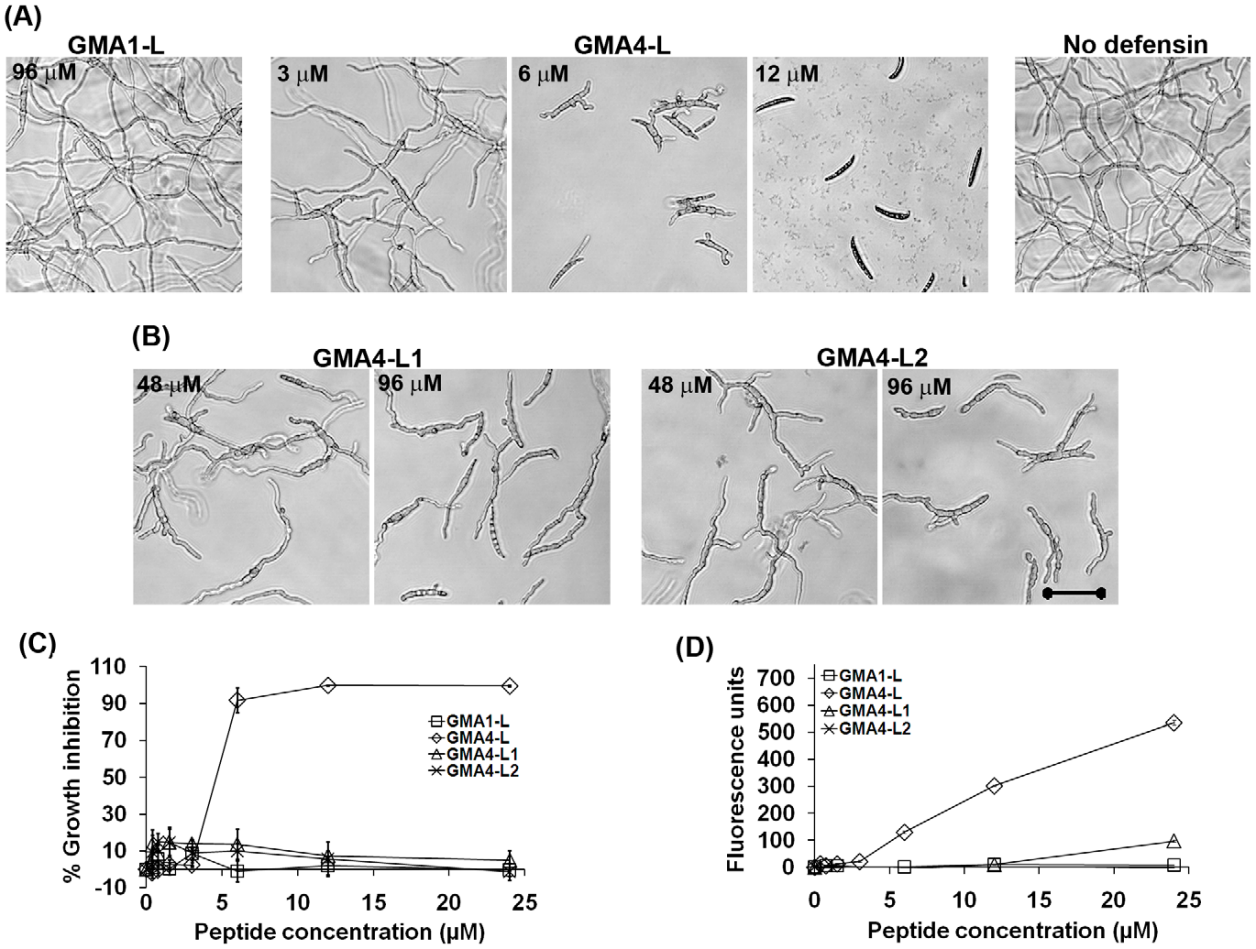
The hexapeptide RGFRRR of the MtDef4 γ-core motif alone is capable of inhibiting fungal growth, but its activity depends on the presence of F_37_ and R_38_ residues. (A) Images showing the inhibition of conidial germination and hyphal growth at different concentrations of GMA1-L with a sequence RDDFR and GMA4-L with a sequence RGFRRR. Images were taken after 16 hours of incubation of PH-1 conidia with peptides. Bar = 50 µm. Note the potent *in vitro* antifungal activity of GMA4-L and complete lack of antifungal activity of GMA1-L, (B) Morphology of inhibition of conidial germination and hyphal growth inhibition at different concentrations of GMA4-L1 and GMA4-L2. Values are means of at least three replications. Error bars indicate standard deviations. (C) Quantitative measurement of the inhibition of fungal growth and (D) SG uptake by the peptides GMA1-L, GMA4-L and its variants GMA4-L1 and GMA4-L2.

The next step was to determine which residues in the MtDef4 γ-core motif are important for antifungal activity against *F. graminearum* (Figure S1). The first peptide tested was GMA4-L (RGFRRR), from within the γ-core motif of MtDef4. GMA4-L peptide carried a net charge of +4 and inhibited fungal growth at a concentration as low as 3 µM (Figure 7A). At a concentration of 6 µM, GMA4-L peptide inhibited 91±7% of fungal growth after 36 hours and at 12 µM, it caused 100% growth inhibition (i.e., no fungal conidial germination) (Figure 7C). As expected, the GMA1-L (RDDFR) peptide, with a net charge of 0, possessed no antifungal activity. These results suggest that the cationicity is a major contributor to the antifungal activity of the peptide (see below) and that the RGFRRR motif plays an important role in the antifungal activity of MtDef4.

We had previously shown that the R38Q mutation in the γ-core markedly reduced the antifungal activity of MsDef1 [15]. Therefore, the importance of the cationic Arg residue at position 4 of GMA4-L (RGFRRR) (corresponding to Arg_38_ of MtDef4) for the antifungal activity of this peptide was determined. In addition, to understand the importance of hydrophobicity in the antifungal activity of the peptide, the hydrophobic Phe residue at position 3 of GMA4-L (corresponding to F_37_ of MtDef4) was also changed. To this end, two variants, GMA4-L1 (RGARRR) and GMA4-L2 (RGFARR), were synthesized and compared for their antifungal activity to that of GMA4-L (RGFRRR). Both variants were much less potent inhibitors of fungal growth than RGFRRR. At 16 hours, both variants at 24 µM caused mild growth inhibition of the fungus, but at 36 hours, no significant reduction in growth inhibition was observed (Figure 7C). Only at a high concentration of 96 µM, moderate (30–40%) growth inhibition was evident at 16 hours (Figure 7B), and this persisted even at 36 hours (Figure S4A). These results indicate that a hydrophobic amino acid F_37_ and a charged residue R_38_ are important for the antifungal activity of the RGFRRR peptide.

GMA1-L, GMA4-L and its variants GMA4-L1 and GMA4-L2 were also compared for their ability to permeabilize the fungal plasma membrane. Only GMA4-L was capable of inducing concentration-dependent increase in SG uptake. GMA4-L1 induced a modest uptake of SG (>100 fluorescence units) only at a concentration 24 µM. GMA1-L and GMA4-L2 failed to induce any uptake of SG at all concentrations tested (Figure 7D).

### Synthetic peptides containing the γ-core motifs plus carboxy-terminal 6 amino acids of MsDef1 and MtDef4 exhibit antifungal activity against other ascomycetous fungi, *F. verticillioides* and *Aspergillus flavus*

**Figure 8:**
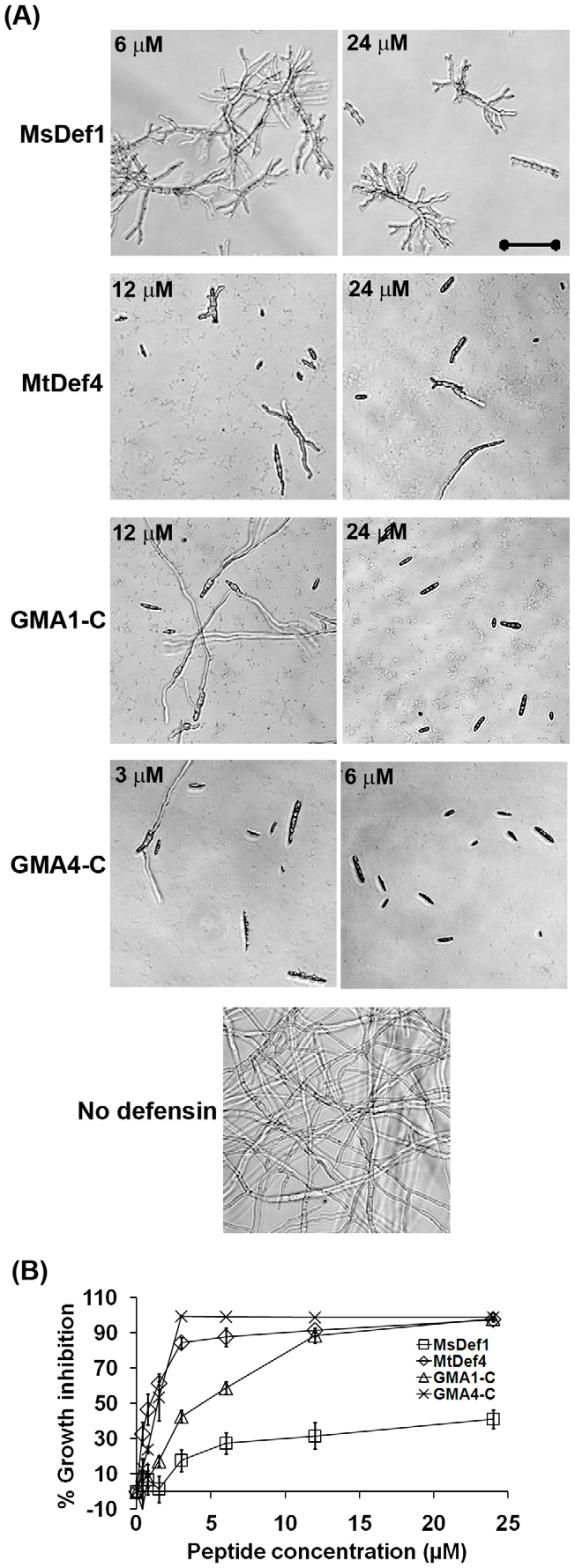
MsDef1, MtDef4 and peptides GMA1-C and GMA4-C inhibit the growth of another ascomycetous fungal pathogen *F. verticillioides* M3125 *in vitro*. (A) Images showing the inhibition of *F. verticillioides* conidial germination and hyphal growth and (B) Quantitative measurement of the inhibition of fungal growth at different concentrations of MsDef1, MtDef4 and the peptides, GMA1-C and GMA4-C. Images were taken after 16 hours of incubation of M3125 microconidia with proteins/peptides. Bar = 50 µm. Values are means of at least three replications. Error bars indicate standard deviations.

**Figure 9:**
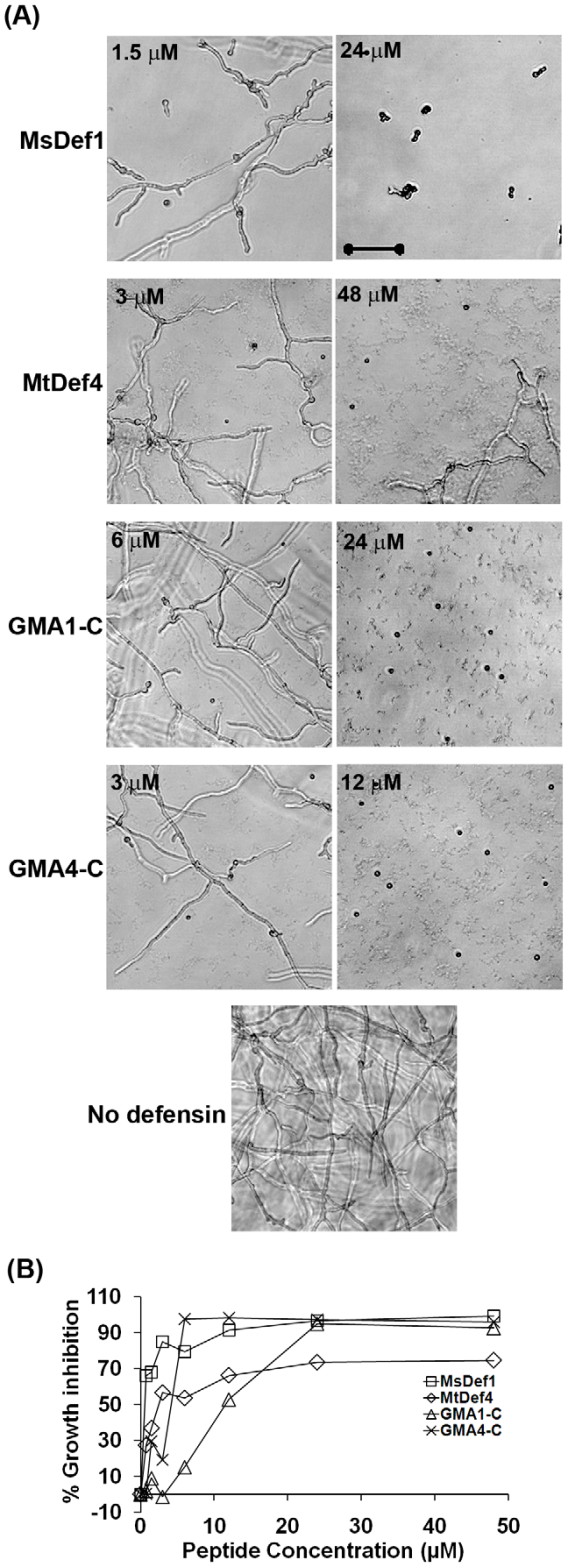
Antifungal activity of MsDef1, MtDef4 and derived peptides against an ascomycetous pathogen *Aspergillus flavus*. (A) Images showing the inhibition of *A. flavus* conidial germination and hyphal growth and (B) Quantitative measurement of the inhibition of fungal growth at different concentrations of MsDef1, MtDef4 and the peptides, GMA1-C and GMA4-C. Images were taken after 16 hours of incubation of *A. flavus* conidiospores with proteins/peptides. Bar = 50 µm. Values are means of at least three replications. Error bars indicate standard deviations.

Since one of the main objectives of understanding the structure-activity relationships of antimicrobial peptides is to explore their biotechnological applications, the activity of MsDef1, MtDef4 and the synthetic peptides GMA1-C and GMA4-C was tested against two other economically important ascomycetous mycotoxigenic fungi, *F. verticillioides* and *A. flavus*. *F. verticillioides* causes *Fusarium* ear rot disease in maize, whereas *A. flavus* causes *Aspergillus* rot in this crop as well as in peanut and cotton. They both produce mycotoxins that significantly impact the safety of the food products derived from the infected seed of these crops. In addition, the morphology of the conidia of these two fungi is significantly different from that of the conidia of *F. graminearum*. Both GMA1-C and GMA4-C exhibited antifungal activity against these fungi; however, there were significant differences in their potency. Importantly, *F. verticillioides* was much less sensitive to full-length MsDef1 than to GMA1-C (Figure 8A). At 24 µM, GMA1-C inhibited more than 90% of the fungal growth, while MsDef1 caused only 41±5% growth inhibition. Like *F. graminearum*, *F. verticillioides* also displayed the hyperbranching phenotype in the presence of MsDef1, but not in the presence of GMA1-C. Even GMA4-C had more potent antifungal activity than the full-length MtDef4, but the difference in the activity was not as significant as that observed between MsDef1 and GMA1-C (Figure 8B). Unlike *Fusarium* spp., *A. flavus* was more sensitive to MsDef1 than MtDef4 (Figure 9A). Although conidia germinated in the presence of 6 to 48 µM MsDef1, the germ tubes and hyphal extension were severely affected thus leading to more than 90% growth inhibition at concentrations of 12 to 48 µM. Furthermore, no hyperbranching phenotype was observed in *A. flavus* (Figure 9A). Most of the conidia did not germinate in the presence of 12 to 48 µM of MtDef4, ultimately resulting in a growth inhibition of approximately 75% at 48 µM. Full-length MsDef1 was however more active against *A. flavus* than GMA1-C. In contrast, GMA4-C was more active than the full-length MtDef4 (Figure 9B). These results have significant implications for the use of these peptides for genetic engineering of fungal resistance in crops.

## Discussion

In this study, we have studied the structure-activity relationships of two antifungal plant defensins MsDef1 and MtDef4 that exhibit different modes of antifungal action [16], [17]. Both proteins inhibit the growth of *F. graminearum* at micromolar concentrations, but induce strikingly different morphological responses in the fungus. It was therefore of interest to determine the structural motifs and amino acid sequences contributing to the potent antifungal activity of these proteins and elucidate requirements for the morphogenicity of MsDef1. We previously showed that the major determinants of the antifungal activity of MsDef1 resided in the carboxy-terminal region spanning amino acid 31 to 45 [15]. Closer examination of this sequence and the corresponding sequence of MtDef4 revealed the presence of a highly conserved γ-core motif, the hallmark feature of the three-dimensional structure of disulfide-containing antimicrobial peptides from evolutionarily diverse organisms [22]. The γ-core motif of both defensins satisfied the consensus sequence GXC(X_3–9_)C and were part of the predicted three-dimensional solvent-exposed β2–β3 loop region of each protein [20], [28], [29], [30], [31], [32]. Plant defensins also contain the α-core motif with a consensus sequence GXC(X_3–5_)C (Figure 1). This motif however is not conserved in all disulfide-containing antimicrobial peptides and does not always share the characteristic features of the γ-core motif.

The γ-core motifs of MsDef1 and MtDef4 differed in their primary amino acid sequence. The γ-core sequence GRCRGFRRRC of MtDef4 contains five basic amino acids and no acidic amino acids. In comparison, the γ-core sequence GRCRDDFRC of MsDef1 was one amino acid shorter and contained two basic and two acidic amino acids. Both γ-core motifs however shared a hydrophobic Phe residue. The substitution of the γ-core motif of MsDef1 with that of MtDef4 not only enhanced the antifungal activity of MsDef1 more than 2-fold, but also transformed it into a nonmorphogenic defensin. This indicates that major determinants controlling antifungal potency as well as morphogenicity reside within the γ-core motif of each defensin. However, it is also clear that this motif by itself does not contain all requirements for the morphogenic effects of MsDef1 since the GMA1-C peptide, containing this motif plus the carboxy-terminal 6 amino acids, failed to induce hyperbranching of fungal hyphae. Our previous study demonstrated that the amino-terminal 15 residues also had some contribution to the antifungal activity of MsDef1 [15]. It is therefore likely that sequences outside the γ-core motif of MsDef1 are also required to produce the observed morphological changes in the fungal growth (Figure 3). The 15 amino-terminal residues of MsDef1 may contain additional determinants of antifungal activity and thus, together with γ-core motif, may exhibit more potent antifungal activity. It will also be interesting to determine if replacement of the γ-core motif of MtDef4 with that of MsDef1 will enable MtDef4 to exhibit morphogenicity and antifungal activity typical of MsDef1. The ΔFg*gcs1* mutant that lacks plasma membrane sphingolipid GlcCer and exhibits resistance to MsDef1 became sensitive to MsDef1-γ4 indicating that the γ-core motif of MsDef1, directly or indirectly, governs its interaction with GlcCer.

That the antifungal activity of MsDef1 and MtDef4 is concentrated largely in the β2–β3 strands and the interposed loop was further confirmed by our observation that chemically synthesized peptides, GMA1-C and GMA4-C, containing these γ-core sequences plus the carboxy-terminal 6 amino acids of each defensin exhibited strong *in vitro* antifungal activity against *F. graminearum*. Similar results were obtained previously with the radish defensin, RsAFP2, whose antifungal activity also resides mainly in the β2–β3 loop which contains the predicted γ-core motif of this defensin [33], [34]. Interestingly, the homology model of MsDef1 and the NMR structure of a plant defensin NaD1 had also previously predicted a putative effector site in their β2–β3 loop regions [20]. It is worth noting however that the antifungal activity of GMA1-C or GMA4-C was less potent than that of native defensin again confirming that some determinants of antifungal activity reside outside the γ-core motifs. Although chemically synthesized GMA1-C and GMA4-C peptides each contained four cysteines, molecular mass of these peptides indicated no formation of a non-native disulfide bond formation. The possibility that non-native disulfide bonds might have been formed during the interaction of these peptides with the fungus could not be ruled out.

Further studies were carried out to delineate the minimal amino acid sequence required for the antifungal activity of the active regions of MsDef1 and MtDef4. Interestingly, the MtDef4 γ-core motif, but not the MsDef1 γ-core motif, alone possessed antifungal activity. This is consistent with earlier reports on the antimicrobial activity of the γ-core motif of other antimicrobial peptides. Thus, the γ-core motifs of protegrin-1, tachyplesin-1 and RTD-1 alone have been reported to be sufficient for antimicrobial activity [22]. The lack of antifungal activity of the γ-core motif of MsDef1 alone suggests that a complete β3-strand which includes the carboxy-terminal 6 amino acids is needed for antifungal activity. Not surprisingly, highly cationic RGFRRR sequence within the γ-core of MtDef4 exhibited antifungal activity at a concentration as low as 6 µM, but RDDFR sequence within the γ-core of MsDef1 was inactive even when used at 96 µM. Previously, another hexapeptide HKCICY derived from the β3-strand of the plant defensin RsAFP2 was also shown to be active against *F. culmorum* [33]. Recently, PAFs, a group of hexapeptides, derived from a peptide combinatorial library have been shown to have antifungal activity against certain filamentous fungi [35], [36]. One of the peptides, PAF26, containing the sequence RKKWFW also exhibits preferential activity against filamentous fungi [37]. Like PAF26, RGFRRR peptide also contains mainly basic and hydrophobic amino acids. The RGFRRR mutagenesis studies presented here clearly indicated that changing the hydrophobic Phe residue at position 3 or the basic Arg residue at position 4 of this peptide to Ala dramatically reduced the antifungal activity of this peptide. This demonstrates the importance of both hydrophobic and basic residues in the antifungal activity of a defensin. The net charge and IC_50_ values of peptides used in this study (Table 1) lend further support to our findings. More studies are required to determine the relative contribution of other residues to the antifungal potency of this peptide and of the native protein.

Many antimicrobial peptides have been reported to cause permeabilization of plasma membrane. Antifungal plant defensins have also been shown to cause plasma membrane damage in their target fungi as demonstrated by the uptake of fluorescent dyes such as SG or propidium iodide in defensin-challenged fungal cells [25], [26], [38], [39]. The mechanisms by which defensins permeabilize fungal plasma membrane are not fully understood, although it has been recently reported that the permeabilization of fungal hyphae by the plant defensin NaD1 occurs through a cell wall dependent process [39]. In this study, we have found that both MsDef1 and MtDef4 permeabilize the plasma membrane of *F. graminearum*, but their kinetics of permeabilization is significantly different. MtDef4-induced increase in SG uptake was concentration dependent, but MsDef1-induced SG uptake was not. Consistent with its more potent antifungal activity, MtDef4 was more effective in causing membrane permeabilization than MsDef1. Surprisingly, MsDef1-γ4 variant with antifungal potency much higher than that of MsDef1 was also as ineffective as MsDef1 indicating that the ability to permeabilize plasma membrane is not a direct indicator of a peptide's antifungal potency. Hexapeptide RGFRRR caused significant permeabilization of the membrane (Figure 7D) whereas its variant peptides (RGARRR and RGFARR) with reduced activity were ineffective in permeabilizing the fungal plasma membrane (Figure S4B). It has been suggested that hexapeptide is too short to span the fungal plasma membrane as a monomer, requiring perhaps higher-order interactions among monomers to cause membrane permeabilization [37].

There is an interesting correlation between the cationicity and antifungal activity among the six synthetic peptides GMA1-C, GMA4-C, GMA1, GMA4, GMA1-L and GMA4-L, that were synthesized based on full-length MsDef1 and MtDef4 sequences. GMA4-C and GMA4 with the highest cationicity of +5 exhibited the maximum antifungal activity followed by GMA4-L (+4) and GMA1-C (+3). The peptides GMA1 with a net charge of +1 and the uncharged GMA1-L were inactive. The replacement of Phe in GMA4-L (+4) with Ala resulted in significant decrease in antifungal activity though the net charge of the peptide remained unchanged. We suspect that the loss of antifungal ability may be due to a decrease in hydrophobicity resulting from Phe to Ala substitution. The two variants of GMA4-L, GMA4-L1 (+4) and GMA4-L2 (+3) exhibited similar antifungal activity even though they differ in net charge. It may be that the loss in basic residue in GMA4-L2 is compensated for by the presence of a highly hydrophobic Phe. Recent studies involving α-helical antimicrobial peptide D-V13K revealed that antifungal activity increased with an increase in hydrophobicity in the Ascomycota fungi [40], [41]. The model fungus in our studies, *F. graminearum* belongs to Ascomycota and we observed that the increase in hydrophobicity could compensate for the decrease in cationicity. Based on existing information and *in vitro* antifungal studies using *F. graminearum* as a model in this study, we propose that there is a correlation between net positive charge of the peptide and its antifungal activity; however, this correlation seems to be dependent on hydrophobicity. Further, the specific composition of amino acids in the β2–β3 loop region, which manages the distribution of charge and hydrophobicity, in conjunction with the adjacent structural domains defines the specificity and potency of an antifungal defensin. MsDef1, MtDef4 and the peptides (GMA4-C and GMA1-C) derived from each defensin also exhibit *in vitro* antifungal activity against other economically important ascomycetous fungal pathogens, *F. verticillioides* and *A. flavus*. However, there are significant differences in the responses of these fungi to the native defensins and the shorter peptides indicating some degree of specificity in recognition of the γ-core motif by each fungus. It is also likely that the shorter peptides derived from native MsDef1 and MtDef4 might have different molecular targets thus leading to varied activity against *F. verticillioides* and *A. flavus*. Further studies are needed to unravel the shared as well as unique structure-activity relationships of these two defensins.

## Materials and Methods

### Fungal cultures and growth medium

The fungal strains, *F. graminearum* PH-1 and *Δ*Fg*gcs1*, *F. verticillioides* M3125 and *A. flvaus* NRRL 3357 were stored in 20% (v/v) glycerol at −80°C and were routinely cultured on complete medium [42]. For macroconidia (conidia) production, the fungal strains from agar plates were inoculated into 10–25 mL of carboxymethyl cellulose medium [43] and cultured for 2–4 days under shaking conditions at 28°C.

### Protein purification and peptide synthesis

MsDef1 and MtDef4 were expressed in *Pichia pastoris* and purified as described previously [15]. These partially purified defensins were further purified by reverse-phase HPLC column chromatography as described below. A synthetic gene encoding the MsDef1-γ4 substitution variant was obtained from GenScript Corporation (Piscataway, NJ) and subsequently cloned into pET28a and expressed in the Rosetta (DE) strain of *E. coli* as described elsewhere [44]. The MtDef4-γ4 variant was further purified by HPLC (Beckman Coulter, Brea, CA) using a reverse phase C18 column (Deltapak Wat 011793, 150×3.9 mm, 5 µM, 300 A) to obtain >95% purity. Peptides derived from each defensin (Table 1) were synthesized at Genemed Synthesis, Inc (San Antonio, TX). All peptides were purified to >95% homogeneity by reverse phase HPLC and characterized by mass spectroscopy. Concentrations of MsDef1, MtDef4, MsDef1-γ4 and MsDef1-R38Q were determined by BCA assay kit (Pierce, Rockford, IL). Peptide concentrations were determined by quantitative amino acid analysis performed at the Proteomics and Mass Spectrophotometry Facility at the Danforth Center. Defensins as well as peptides were dissolved in sterile ddH_2_O and filtered through 0.22 µM syringe filter before using for *in vitro* antifungal activity assays.

### Homology-based three dimensional structures of MsDef1 and MtDef4

The models shown in Figure 1 were created using the I-TASSER web site for protein structure and function predictions (http://zhanglab.ccmb.med.umich.edu/I-TASSER/) [45], [46], [47]. For this application, the amino acid sequences of MsDef1 and MtDef4 were threaded onto the known structure of a floral defensin NaD1 from *Nicotiana alata* (PDB accession, 1MR4) [20]. The correlation scoring (C-score) in I-TASSER can range from −5 to 2 with higher scores representing greater confidence in the threaded model. For both the MsDef1 and MtDef4 models, the C-score was >1.6, with an estimated RMSD of 0.5 Å+/−0.5 Å. The threaded models had all but one of the expected disulfide bonds, but these were easily fixed by simple rotamer rotations of the cysteine side chains.

### *In vitro* antifungal activity determination

*In vitro* antifungal assays were performed using synthetic fungal medium (SFM) without calcium as described previously [15], [48]. Bight-field images were taken using the transmitted light channel in a Zeiss LSM 510 META confocal microscope to monitor the early visible phenotypic effects of defensins on conidial germination and growth of fungal hyphae (at 16 hours after treatment with defensins). Unless specified otherwise, the quantitative fungal growth inhibition by defensins and synthetic peptides was estimated at 36 hours by measuring the absorbance at 595 nm using Spectramax M2 spectrophotometer (Molecular Devices, Sunnyvale, CA) [48].

### Hyphal tip morphology studies

*F. graminearum* conidia (5×10^4^) were germinated in 400 µL (in each well) of 2× SFM in a 24-well plate with a sterile cover slip at the bottom. After 16–18 hours, hyphae adhered to cover slip were treated with complete inhibitory concentrations of MsDef1, MtDef4, MsDef1-γ4 (Table 1) or sterile water. Hyphae were observed under wide-field microscope after 4 hours and 7–8 hours and images were taken at 60× magnification.

### SYTOX Green uptake assay

For SYTOX Green (Invitrogen, Carlsbad, CA) assay, macroconidia (5×10^4^) were incubated in 50 µL of 2× SFM in a black polystyrene 96-well plate (Corning Inc., Corning, NY) for 16 hours at room temperature. After 16 hours, mixtures of proteins/peptides (at concentrations of 6, 3, 1.5, 0.75, 0.375, 0.19 and 0.1 µM) plus SYTOX Green (0.5 µM final concentration) were added to the hyphae in 50 µL volumes. Assay plates were incubated in dark and the fluorescence was monitored (488 excitation; 540 emission (530 cut-off)) using Spectramax M2 spectrophotometer. Samples containing only SYTOX Green (without defensins) and samples without both defensin and SYTOX Green were used as negative controls. No fluorescence was emitted by hyphae without both defensin and SYTOX Green; fluorescence emitted by sample with just SYTOX Green (without defensins) was considered as background and these fluorescence units were subtracted from samples with defensins before plotting graphs. Black polystyrene plates were used to prevent cross transfer of fluorescence. To confirm the fluorescence measurements, hyphae were also visualized under a Zeiss LSM 510 META confocal microscope with a BPIR filter (excitation wavelength 500–550).

## Supporting Information

Figure S1 **Amino acid sequence alignment of the MsDef1 and MtDef4 homologs from different plants.** Amino acid sequences of MsDef1 homologs (A) and MtDef4 homologs (B) were obtained from NCBI database and were aligned using CLUSTAL W. The α-core and γ-core motifs containing highly conserved GXC(X_3–9_)C consensus are indicated in bold letters. *denotes the only defensin homolog of MtDef4 that does not have the conserved G in the α-core motif.
(TIF)

Figure S2 **Fluorescence image of SG uptake by hyphae treated with different concentrations of MsDef1 and MtDef4.** Note a significant concentration-dependent increase in the uptake of SG induced by MtDef4, but not by MsDef1. Bar = 50 µm. Inset images are white light images.
(TIF)

Figure S3 **Images showing the inhibition of conidial germination and hyphal growth at different concentrations of GMA1-C and GMA4-C.** Both the peptides have antifungal activity but GMA4-C is more potent compared to GMA1-C. Images were taken after 16 hours of incubation of PH-1 conidia with peptides. Bar = 50 µm,
(TIF)

Figure S4 **Antifungal activity and fungal plasma membrane permeabilization induced by the GMA4-L variants, GMA4-L1 and GMA4-L2.** Quantitative measurement of the (A) fungal growth inhibition and (B) SG uptake by fungal hyphae treated with GMA4-L1 and GMA4-L2.
(TIF)
