# Supplementary figures and images for: Structure-Activity Determinants in Antifungal Plant Defensins MsDef1 and MtDef4 with Different Modes of Action against Fusarium graminearum
Source: PLoS One. 2011 Apr 13;6(4):e18550. doi: 10.1371/journal.pone.0018550 (PMC3076432; doi:10.1371/journal.pone.0018550)

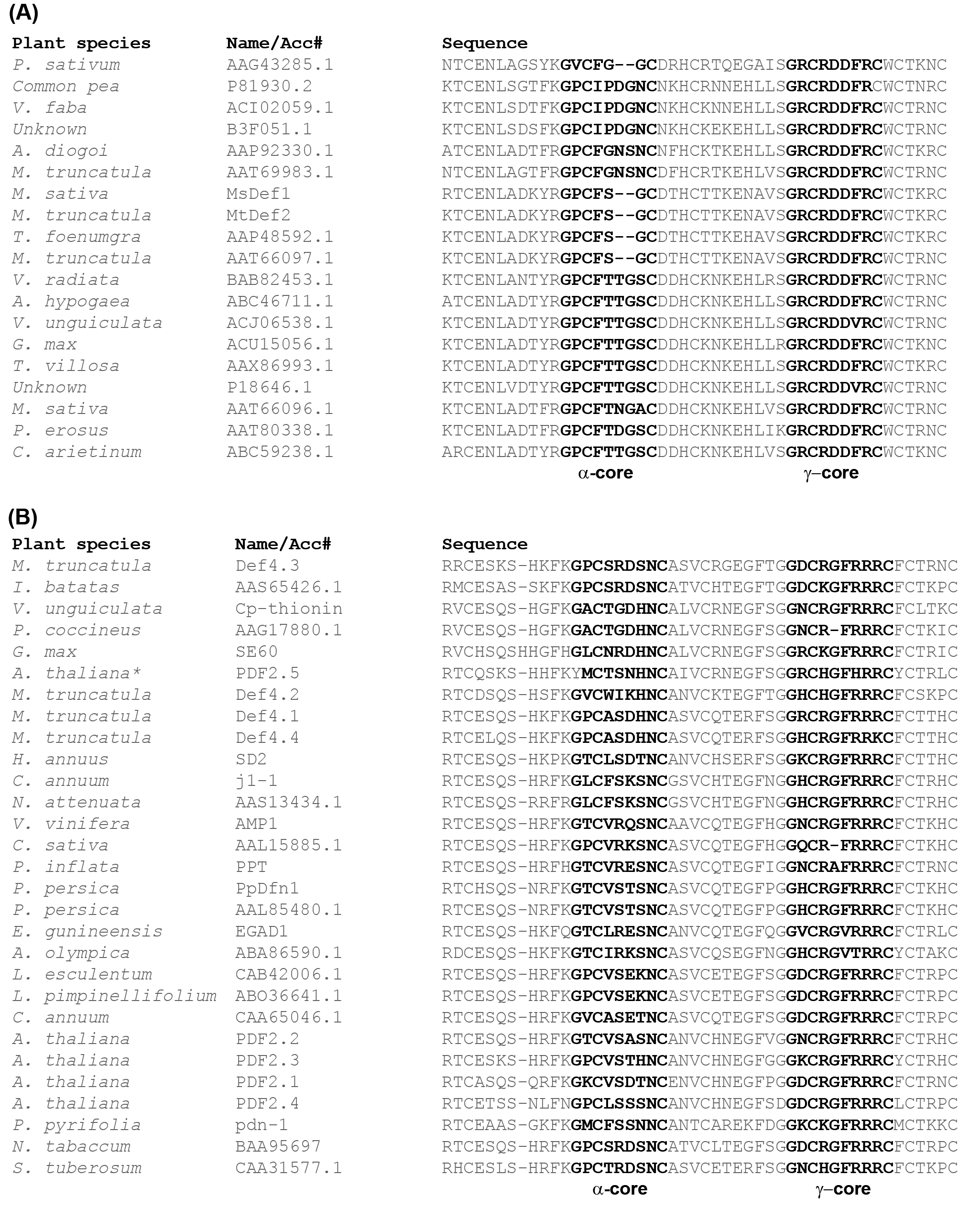

Supplement: Figure S1 — Amino acid sequence alignment of the MsDef1 and MtDef4 homologs from different plants. Amino acid sequences of MsDef1 homologs (A) and MtDef4 homologs (B) were obtained from NCBI database and were aligned using CLUSTAL W. The α-core and γ-core motifs containing highly conserved GXC(X3–9)C consensus are indicated in bold letters. *denotes the only defensin homolog of MtDef4 that does not have the conserved G in the α-core motif. (TIF) [file pone.0018550.s001.tif]

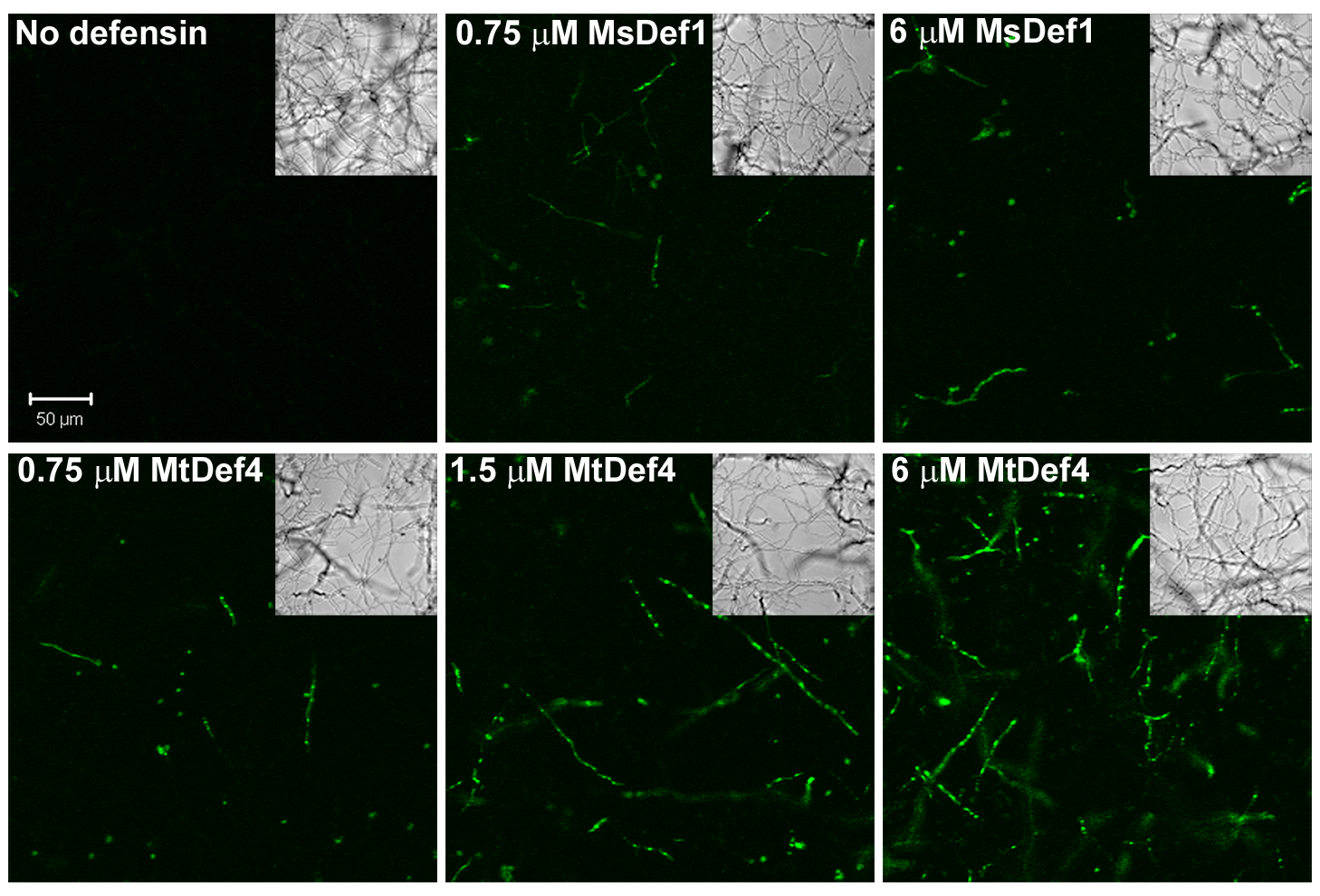

Supplement: Figure S2 — Fluorescence image of SG uptake by hyphae treated with different concentrations of MsDef1 and MtDef4. Note a significant concentration-dependent increase in the uptake of SG induced by MtDef4, but not by MsDef1. Bar = 50 µm. Inset images are white light images. (TIF) [file pone.0018550.s002.tif]

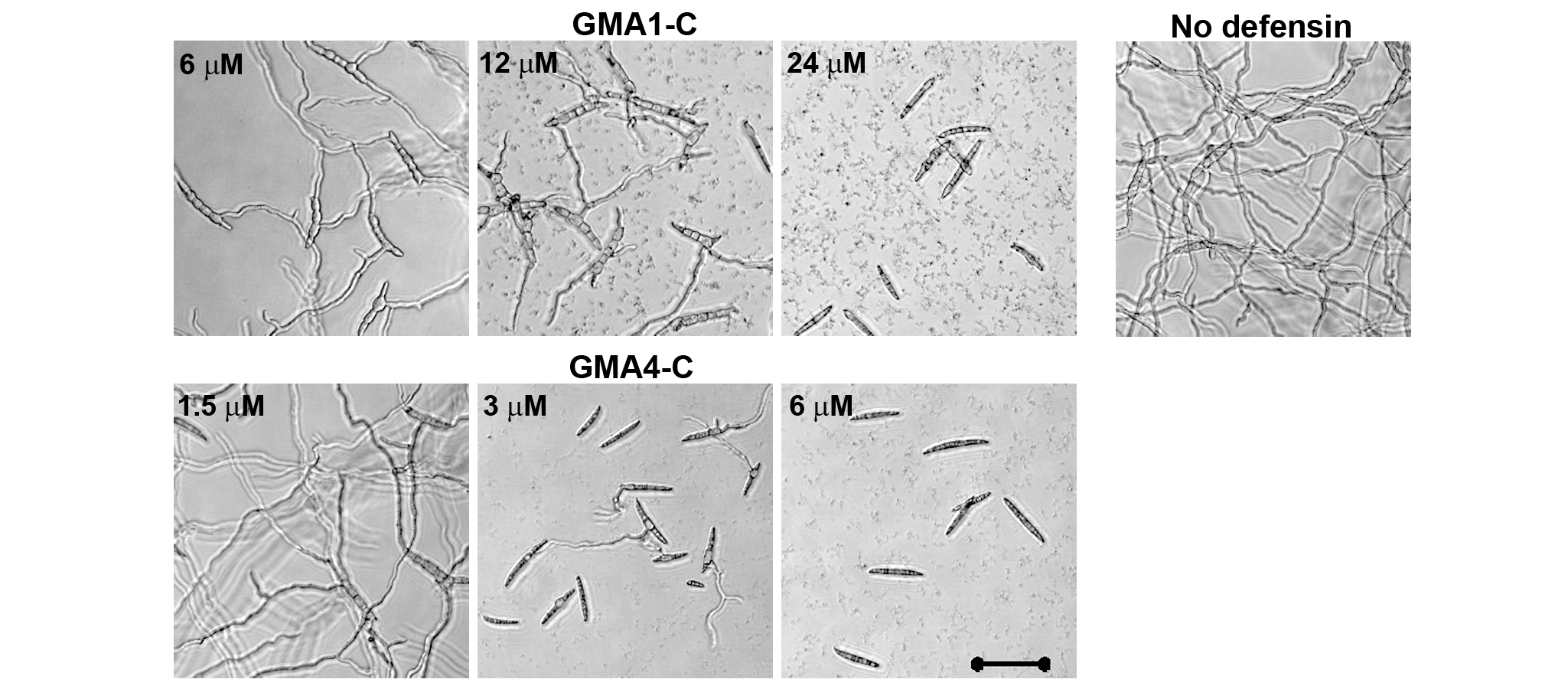

Supplement: Figure S3 — Images showing the inhibition of conidial germination and hyphal growth at different concentrations of GMA1-C and GMA4-C. Both the peptides have antifungal activity but GMA4-C is more potent compared to GMA1-C. Images were taken after 16 hours of incubation of PH-1 conidia with peptides. Bar = 50 µm, (TIF) [file pone.0018550.s003.tif]

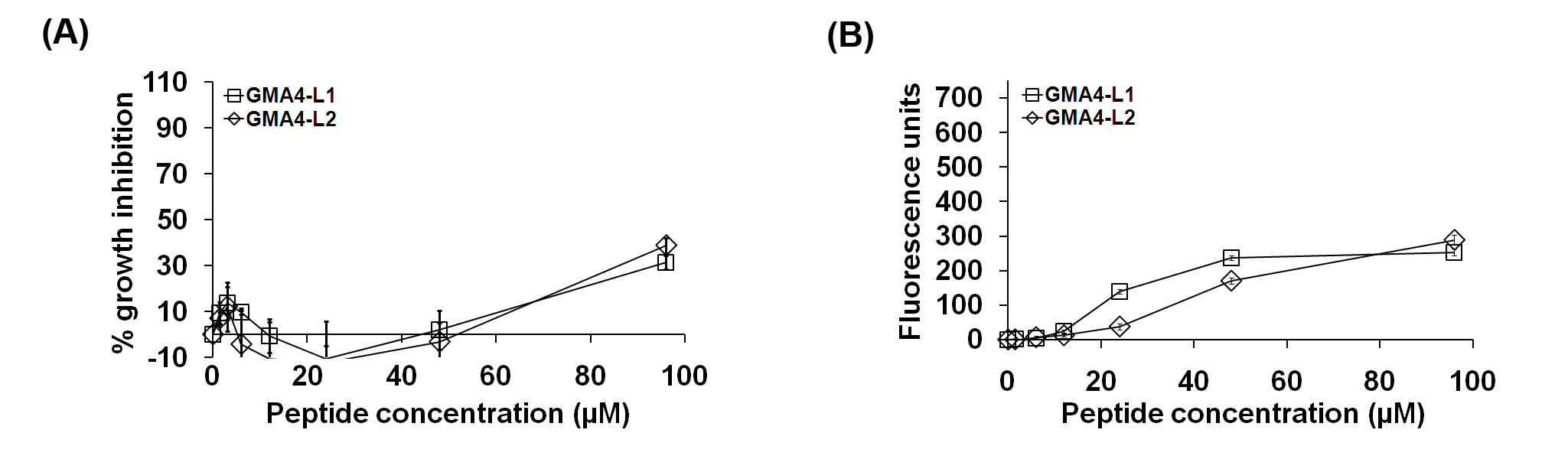

Supplement: Figure S4 — Antifungal activity and fungal plasma membrane permeabilization induced by the GMA4-L variants, GMA4-L1 and GMA4-L2. Quantitative measurement of the (A) fungal growth inhibition and (B) SG uptake by fungal hyphae treated with GMA4-L1 and GMA4-L2. (TIF) [file pone.0018550.s004.tif]
